# Long Lasting Protein Synthesis- and Activity-Dependent Spine Shrinkage and Elimination after Synaptic Depression

**DOI:** 10.1371/journal.pone.0071155

**Published:** 2013-08-09

**Authors:** Yazmín Ramiro-Cortés, Inbal Israely

**Affiliations:** 1 Champalimaud Neuroscience Programme at Instituto Gulbenkian de Ciência, Oeiras, Portugal; 2 Champalimaud Neuroscience Programme, Champalimaud Centre for the Unknown, Lisbon, Portugal; Instituto de Medicina Molecular, Portugal

## Abstract

Neuronal circuits modify their response to synaptic inputs in an experience-dependent fashion. Increases in synaptic weights are accompanied by structural modifications, and activity dependent, long lasting growth of dendritic spines requires new protein synthesis. When multiple spines are potentiated within a dendritic domain, they show dynamic structural plasticity changes, indicating that spines can undergo bidirectional physical modifications. However, it is unclear whether protein synthesis dependent synaptic depression leads to long lasting structural changes. Here, we investigate the structural correlates of protein synthesis dependent long-term depression (LTD) mediated by metabotropic glutamate receptors (mGluRs) through two-photon imaging of dendritic spines on hippocampal pyramidal neurons. We find that induction of mGluR-LTD leads to robust and long lasting spine shrinkage and elimination that lasts for up to 24 hours. These effects depend on signaling through group I mGluRs, require protein synthesis, and activity. These data reveal a mechanism for long lasting remodeling of synaptic inputs, and offer potential insights into mental retardation.

## Introduction

Changes in synaptic weight have been proposed to serve as the physiological basis for learning and memory [1], and the production of new proteins has been shown to be critical for such long lasting processes [2], [3]. These modifications can result in either potentiated or depressed synaptic transmission at individual synapses. The induction of long-term potentiation (LTP) corresponds with growth of new spines, the sites where the majority of excitatory synapses are located on neuronal dendrites, indicating that activity may physically alter neuronal connectivity [4], [5]. When an individual spine is potentiated through glutamate uncaging, such activity promotes an increase both in the current amplitude and the volume of that stimulated spine [6], [7]. Interestingly, when new protein synthesis is triggered, activity can lead to the facilitation of plasticity at other locations, allowing for the integration of information [8]–[10]. Similarly, synaptic and structural plasticity induced at the level of individual inputs may influence the expression of plasticity at neighboring spines [9], [11]. Specifically, activity that leads to new protein synthesis can facilitate the expression of plasticity at other sites for up to 1.5 hours and as far as 70 µm away [9]. This cooperation between individual sites demonstrates a prolonged period over which dendritic integration of information can occur when new proteins are available. Alternatively, such potentiation can also lead to competition for the expression of plasticity when simultaneously induced at multiple sites, resulting in bidirectional changes in the volumes of those spines [9]. These observations indicate that mechanisms exist not only for the regulation of spine growth, but also for spine shrinkage.

While the structural correlates of LTP have begun to be elucidated, those occurring in response to long lasting synaptic depression are less understood. Long-term depression (LTD) can lead to the production of new proteins, although relatively little is known about the structural modifications resulting from these changes in efficacy. Synaptic depression can be induced either through activation of NMDA receptors (NMDARs) or through metabotropic glutamate receptors (mGluRs) [1], [12]–[14]. However, these forms of LTD have different biochemical mechanisms of induction and expression, and in particular, NMDAR-mediated LTD does not require new protein synthesis [1], [13]–[17]. This difference has a potentially significant impact on the structural correlates of synaptic depression. While NMDA mediated LTD results in the shrinkage of spines [18]–[21], it is unclear whether these changes are long lasting or widespread. In the case of synaptic potentiation for example, protein synthesis independent plasticity occurs preferentially at smaller spines [6], and leads to short term structural modifications (∼1.5 h), while long lasting, protein synthesis dependent stimuli lead to correspondingly longer lasting structural changes (>4 h) on spines of various sizes [9]. Indeed, it seems that NMDAR-LTD preferentially shrinks smaller spines [20]. Given the importance of protein synthesis for long lasting changes in synaptic weights, we wanted to determine if this form of long lasting plasticity would lead to long lasting structural changes at various inputs. mGluR dependent LTD leads to robust induction of, and is dependent upon, new protein synthesis [22], [23]. Although this form of LTD has been studied at individual spines (Holbro *et al.*, 2009), the structural correlates of such long lasting plasticity are unknown. Interestingly, impairments in mGluR mediated plasticity have been implicated in the mental retardation syndrome Fragile X [24], which is associated with increases in spine density [25], [26]. These structural alterations have also been observed in mice lacking mGluR5 [27]. Therefore, understanding how long lasting, protein synthesis dependent synaptic depression affects dendritic spines may provide insights as to the causes of cognitive dysfunction.

Here, we investigate the structural correlates of mGluR mediated LTD using live two- photon imaging of dendritic spines in hippocampal pyramidal neurons. We find that global induction of LTD, through a brief application of the mGluR1/5 agonist DHPG, leads to the robust shrinkage and elimination of spines. These changes are long lasting, and could be observed for up to 24 hours. Interestingly, this occurs irrespectively of initial spine size. Furthermore, spine shrinkage and elimination require new protein synthesis as well as synaptic activity, and is independent of NMDA receptors. Elucidating this mechanism contributes to our understanding of the learning rules governing bidirectional changes in synaptic plasticity and structure, which may play a critical role in shaping the organization of inputs within the dendritic tree.

## Materials and Methods

### Ethics Statement

All animal experiments were carried out in accordance with European Union regulations on animal care and use, and with the approval of the Portuguese Veterinary Authority (DGV).

### Mouse Hippocampal Organotypic Slice Cultures

Cultured hippocampal slices were prepared from postnatal day 7–10 C57BL/6J mice [28]. Briefly, 350 µm thick slices were made with a chopper in ice-cold ACSF containing 2.5 mM KCl, 26 mM NaHCO_3_, 1.15 mM NaH_2_PO_4_, 11 mM D-glucose, 24 mM sucrose, 1 mM CaCl_2_ and 5 mM MgCl_2_, and cultured on membranes (Millipore). The slices were maintained in an interface configuration with the following media: 1× MEM (Invitrogen), 20% horse serum (Invitrogen), GlutaMAX 1 mM (Invitrogen), 27 mM D-glucose, 30 mM HEPES, 6 mM NaHCO_3,_ 1 M CaCl_2_, 1 M MgSO_4_, 1.2% ascorbic acid, 1 µg/ml insulin. The pH was adjusted to 7.3, and osmolarity adjusted to 300–310 mOsm. All chemicals were from Sigma unless otherwise indicated.

### Transfection

Hippocampal neurons from organotypic slice cultures were transfected using a Helios gene gun (Bio-Rad) after 6–7 days *in vitro* (DIV). Gold beads (10 mg, 1.6 µm diameter, Bio-Rad) were coated with 100 µg Dendra-2C plasmid DNA (Evrogen) according to the manufacturer’s protocol and delivered biolistically into the slices at 180 psi. Experiments were performed 2–4 days post-transfection.

### Two-photon Imaging and Spine Volume Determination

Two-photon imaging was performed using a galvanometer-based scanning system (Prairie Technologies) on a BX61WI Olympus microscope, using a Ti:sapphire laser (910 nm for imaging Dendra-2C; Coherent) controlled by PrairieView software. Slices were perfused with oxygenated ACSF containing 127 mM NaCl, 2.5 mM KCl, 25 mM NaHCO_3_, 1.25 mM NaH_2_PO_4_, 25 mM D-glucose, 2 mM CaCl_2_ y 1 mM MgCl_2_ (equilibrated with O_2_ 95%/CO_2_ 5%) at room temperature at a rate of 1.5 ml/min. Imaging was started 45 min to 1 h after slice incubation began. Secondary or tertiary dendrites of CA1 neurons, located approximately 100 µm away from the soma, were imaged using a water immersion objective (60×, 1.0 NA, Olympus LUMPlan FLN) with a digital zoom of 10×. Image stacks (0.3 µm per section, 15 µm total thickness, ∼3 min in duration) were collected once every 5 min for up to 4 hours at a resolution of 1024×1024 pixels, resulting in a field of view approximately 20 µm × 20 µm. In some experiments, such as those lasting 12–24 hours, images were collected once every 30 min for 12 h. Z-stacks were used to quantify spine volumes in all experimental conditions, and all images within an experiment were acquired under the same imaging conditions maintaining equal laser power and PMT gain settings.

One dendritic segment was analyzed per neuron per experiment. For each experiment, all spines with a discernible head within the field of view were included in the analysis, with the exception of spines that were obstructed by other structures, resulting in an average of 9 spines scored per experiment. All experiments included a baseline imaging period of 20 min. Spines that were stable during the baseline, but ceased to be visible sometime after experimental treatments were scored as ‘eliminated’. Spine volume measurements were carried out using FWHM, as previously described [9], as it is a measure that is independent of fluorescence intensity [29]. Briefly, the full width at half maximum (FWHM) of the spine head was measured and used to calculate the volume, based on the volume of a sphere, using FWHM as the diameter. Measures were carried out in ImageJ (NIH) with a custom written plugin that performed image registration, and a best fit analysis of the FWHM for each time point. All normalization was performed on a per spine basis as a percent of the average baseline value for that spine. Results are presented as mean ± SEM using one-way ANOVA. *Post hoc* tests were conducted using Tukey test for means comparisons. A value of *p<*0.001 was accepted to indicate a statistically significant difference.

### LTD Induction

Synaptic depression was induced by bath application of (R*S*)-3,5-dihydroxypheylglycine (DHPG, 50 µM) for 5 min [22]. Some experiments with DHPG were performed in the presence of Tetrodotoxin Citrate (TTX, 0.5 µM) or D(-)-2-Amino-5-phosphonopentatonic acid (D-AP5, 50 µM). DMSO (0.05%) was added to the ACSF to control for trace vehicle present in the Anisomycin (Aniso, 50 µM) conditions. For protein synthesis inhibition experiments, slices were pre-incubated in Anisomycin or Cycloheximide (CHX, 60 µM) for 20 min, and DHPG was added at the end of each pre-incubation period for the stimulation time as previously described. Some experiments were performed in the presence of the group I mGluRs antagonists: LY367385 (100 µM) and 2-Methyl-6-(phenylethynyl) pyridine hydrochloride (MPEP, 10 µM). Antagonists were applied together for 20 min, and DHPG was added in order to induce stimulation [30] as previously described. DHPG, TTX, Anisomycin, Cycloheximide, LY367385 and MPEP were from Tocris.

### Patch Clamp Electrophysiology

Hippocampal slice cultures were pre-incubated for 45 min to 1 h at room temperature and perfused continuously with ACSF. Whole cell voltage-clamp recordings were performed in CA1 pyramidal neurons, using 7–8 MΩ electrodes filled with internal solution containing: 136.5 mM K-gluconate, 9 mM NaCl, 17.5 mM KCl, 10 mM HEPES, 0.2 mM EGTA, pH adjusted to 7.2 with KOH, and 284 mOsm. Cells were voltage clamped at −65 mV. Cellular recordings in which series resistance was higher than 25 MΩ were discarded, and stability was assessed throughout the experiment (±20%). EPSC responses were evoked by stimulation of CA3 Schaffer collateral afferents with a bipolar platinum/iridium stimulating electrode (FHC, Bowdoin, ME). Responses were collected every 30 s using a stimulation intensity (0.1–2 mA) yielding 50–60% of the maximal response. Signals were acquired using a Multiclamp 700B amplifier (Molecular Devices), data was digitized with a Digidata 1440 at 3 kHz. EPSC amplitudes were analyzed using custom software written in Matlab.

### Statistical Analysis

Results are presented as mean ± SEM. using one-way ANOVA. *Post hoc* test were conducted using Tukey test for means comparisons. A value of *p*<0.001 was accepted to indicate a statistically significant difference.

## Results

### mGluR-mediated LTD Promotes Long Lasting Spine Shrinkage and Elimination

**Figure 1:**
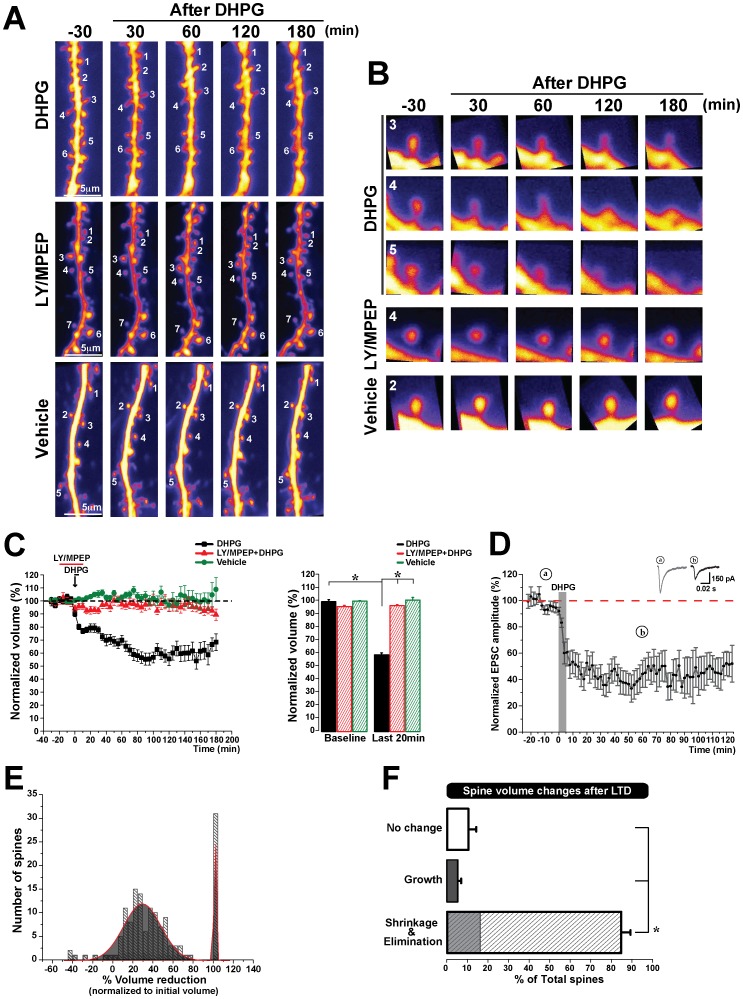
mGluR-LTD leads to spine shrinkage and elimination. **A**) Representative two-photon images (maximum-intensity z-stack projections) of secondary dendrites from CA1 neurons transfected with Dendra-2C before and after LTD induction (DHPG), in presence of mGluR antagonists (LY36735 and MPEP) and control condition (vehicle). **B**) Additional images on the right show representative spines for each condition, the number in the left corner indicate the spine that correspond on panel A. Z-stacks projections were collected once every 5 min for 210 min. **C**) Spine shrinkage was observed for over three hours following LTD induction (black line), but not following LTD in the presence of mGluR antagonists LY367385/MPEP (red line), or in control ACSF (vehicle) conditions (green line). Normalization was performed as a percent of the average baseline volume of each spine. Significant, long lasting shrinkage of spines was observed following LTD induction compared to control conditions (pooled data from 17 cells). No significant shrinkage was induced in the presence of mGluR antagonists (data from 5 cells) or in control conditions with ACSF alone (data from 10 cells). *, *p*<0.001. Error bars indicate mean ± SEM. **D**) LTD was induced in CA1 hippocampal slice cultures with DHPG. EPSC amplitudes were recorded for 2 h in CA1 pyramidal neurons evoked by field stimulation in CA3 before and after induction of LTD. Representative traces during the baseline (**a**) and 60 min after DHPG application (**b**). Normalization performed as percentage of average baseline values for each experiment. **E**) Histogram representing spine shrinkage distributions from across all LTD experiments performed (161 spines, 17 cells). Spines distributed into two peaks, representing a group that reduced in volume and a group that were eliminated. **F**) Quantification of spines that shrink, grow, or do not change after mGluR-LTD induction from pooled data of 161 spines/17 cells. The shaded area within the shrinkage bar corresponds to the percentage of spines which are eliminated. *p<0.001. Error bars indicate means ± SEM.

In order to determine whether protein synthesis dependent synaptic depression leads to structural changes at dendritic spines, we induced a robust form of mGluR mediated LTD through the brief bath application of the group I mGluR agonist DHPG in mouse hippocampal slice cultures [22]. We visualized spine morphology in dendrites using two-photon microscopy of CA1 pyramidal neurons biolistically labeled with Dendra2-C. Following the global induction of mGluR-LTD, we observed a dramatic and significant shrinkage of spines (58.8±1.5% of initial volume, n = 48 spines, 7 cells) compared to controls (100.8±2.0% of initial volume, n = 70 spines, 10 cells) (Fig. 1 A, B, C). Interestingly, the population of spines that showed a decrease in volume distributed into two groups: one that shrank in size and a second that was eliminated (the latter corresponding to 18.6% of all spines that became smaller) (Fig. 1 E). Therefore, across all of the LTD experiments conducted (n = 161 spines, 17 cells), we found that the majority of spines quantified decreased in volume (138 decreased, 8 grew, 15 showed no change, corresponding to 86%, 5%, and 9% of the total, respectively) (Fig. 1 F, total spine shrinkage is shown in the hatched bar, and the grey shaded area within represents eliminated spines), unlike control conditions in which the majority of spines remained stable (9 decreased, 14 grew, 47 showed no change, corresponding to 13%, 20%, and 67% of the total). The stability of the spine volumes observed in the control group for the duration of the experimental period indicated that the hippocampal slice cultures were viable and healthy.

To verify that the observed spine shrinkage was indeed mediated by mGluRs, we induced LTD in the presence of the group I mGluRs antagonists LY367385 (mGluR1) and MPEP (mGluR5). Under these conditions, spines did not show a significant change in volume from their original size (96.7±0.7% of initial volume, n = 53 spines, 5 cells) (Fig. 1 A, B, C). We wanted to confirm that the spine shrinkage observed following DHPG correlated with the induction of synaptic depression in our slice cultures, and thus we performed whole-cell patch electrophysiology (voltage-clamp) recordings. We induced LTD as in the above experiments (DHPG, 50 µM, 5 min) and recorded Excitatory Post Synaptic Currents (EPSCs) from CA1 pyramidal neurons. As previously reported [22], [31], [32], DHPG induced robust long-term depression in CA1 neurons (62.8±2.5%, n = 8 cells) (Fig. 1 D, 2 D). These data indicate that the induction of long lasting, mGluR-mediated LTD leads to robust shrinkage of spines following the onset of synaptic plasticity.

### Spine Shrinkage Mediated by mGluR-LTD Requires Protein Synthesis and Activity, but does not Require NMDARs

**Figure 2:**
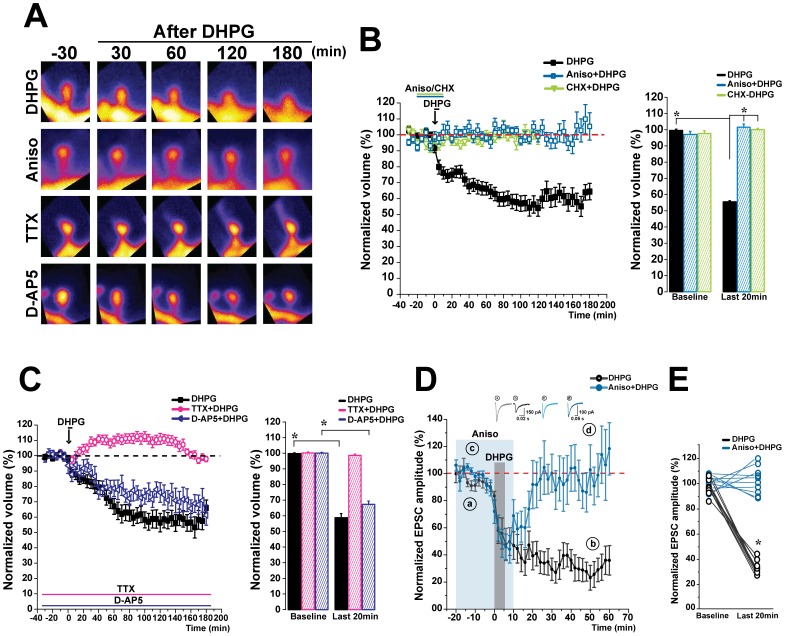
Spine shrinkage promoted by mGluR-LTD is NMDA independent, requires protein synthesis and activity. **A**) Spine shrinkage is only observed when LTD is induced under conditions of normal protein synthesis and activity. Time-lapse two-photon images (z-stack maximum-intensity projections) of representative dendritic spines before and after LTD induction, in the presence Anisomycin, TTX or D-AP5. **B**) No spine shrinkage was observed when LTD was induced in the presence of protein synthesis blockade with either Aniso (blue line) or CHX (green line). Spine volumes were analyzed once every 5 min for over 3 h. Bar graph on the right quantifies the spine volume changes during LTD, compared to LTD induced in the presence of protein synthesis blockade (Aniso in blue or CHX in green). *p<0.001. Error bars indicate means ± SEM. **C**) No spine shrinkage was observed when activity was blocked during LTD induction with TTX (pink line), whereas shrinkage was observed in presence of NMDAR blockade. Time course of spine volume changes during LTD in the presence or absence of TTX or D-AP5. Bar graph quantifies the spine volume changes observed during LTD with or without TTX or D-AP5. Normalization performed as percentage of average baseline values for each spine. *p<0.001. Error bars indicate means ± SEM. **D**) LTD induction in the presence of protein synthesis blockade results in short lasting synaptic depression in hippocampal slice cultures. EPSC amplitudes recorded in CA1 pyramidal neurons evoked by field stimulation in CA3 before and after induction of LTD by DHPG application in presence or absence of Aniso (50 µM/30 min). Representative traces during the baseline (**a**,**c**) and 60 min after DHPG application alone (**b**) or in the presence of Aniso (**d**). **E**) Protein synthesis blockade during LTD induction in hippocampal slice cultures occludes long-term depression. Group data from LTD induced with or without protein synthesis blockade (9 cells per condition). *p<0.001. Error bars indicate means ± SEM.

The expression of mGluR-LTD requires protein synthesis [22]. Therefore, we tested whether the spine shrinkage we observed in response to mGluR-LTD also requires protein synthesis. We induced LTD in the presence of one of either two different protein synthesis inhibitors, Anisomycin (Aniso, a peptidyl transferase inhibitor) or Cyclohexamide (CHX, a blocker of elongation), in order to reduce any potential drug artifacts (Fig. 2 A). As expected, we did not detect spine shrinkage upon mGluR-LTD induction in the presence of either Aniso (101.5±1.9% of initial volume, n = 31 spines, 4 cells, 50 µM, 30 min) or Cycloheximide (100±0.8% of initial volume, n = 30 spines, 3 cells, 60 µM, 30 min), whereas DHPG stimulation again led to robust spine shrinkage (55.5±0.7% of initial volume, n = 48 spines, 4 cells) (Fig. 2 B). In accordance with previous data [22], whole cell recordings performed in the presence of Anisomycin confirmed that protein synthesis is necessary for the induction of DHPG mediated LTD (103.3±3.8% 9 cells, with DHPG+Aniso, compared to 62.8±2.5%, n = 8 cells, with DHPG alone) (Fig. 2 D, E), while acute, short term depression was unaffected (38.2±3.3%) (Fig. 2 D).

We wanted to determine whether activity is also required for the expression of spine shrinkage following mGluR-LTD. Therefore, we induced DHPG-LTD in the presence of Tetrodotoxin (TTX, 0.5 µM), a sodium channel blocker, in order to eliminate the propagation of action potentials. Interestingly, we found that there was no shrinkage of spines when activity was blocked (98.6±0.8% of initial volume, n = 38 spines, 6 cells), compared to the large reduction in spine volume induced by DHPG without TTX (58.7±2.6% of initial volume, n = 49 spines, 6 cells) (Fig. 2 C). Therefore, we wanted to verify that NMDAR activity was not somehow being recruited by the application of DHPG. To confirm that the spine shrinkage induced by DHPG did not involve NMDAR function, we performed some experiments in the presence of the competitive NMDAR antagonist D-AP5. As expected, spine shrinkage was induced by DHPG despite NMDA blockade (67.3±2.0% of initial volume, n = 33 spines, 3 cells) (Fig. 2 A, C). Therefore, although the mechanism for mGluR mediated spine shrinkage requires activity, it does not function via NMDA receptors. Overall, these data demonstrate that the expression of structural plasticity mediated by mGluRs is reliant on new protein synthesis and synaptic activity, and does not require NMDAR dependent signaling.

### Spine Volume Decreases are Independent of Initial Spine Size

**Figure 3:**
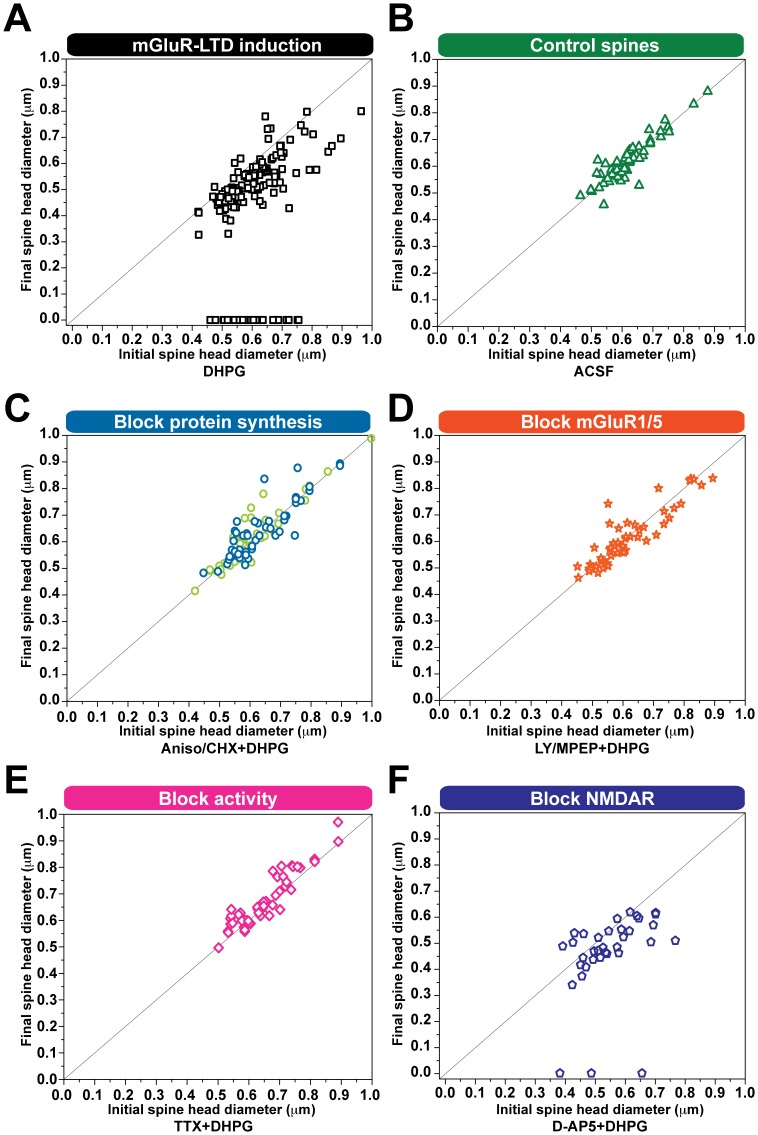
Spine shrinkage and elimination are independent of initial spine size. Two-dimensional plot of initial *versus* final spine head diameter (µm) (15 min before and 2 h after LTD). Spines which fall along the horizontal line, do not show a change in size. **A, F**) Spines of varying initial sizes shrink following mGluR-LTD in absence or presence of D-AP5. **B–E**) Spine sizes remain the same throughout control experimental conditions indicated for each graph.

Structural plasticity resulting from the induction of LTP had been reported to preferentially occur at smaller spines [6]. Subsequently, it has been demonstrated that long lasting forms of synaptic potentiation, which rely on new protein synthesis, can lead to growth of spines of various sizes [9]. Thus, we wanted to determine whether the structural changes following protein synthesis dependent synaptic depression are biased towards occurring at spines of a particular size. We found that irrespective of initial size, both large and small spines were capable of reducing in volume upon mGluR-mediated LTD, either when the initial and final diameter of the spine head was compared (Fig. 3 A, F) or when the change in volume per spine was compared (Fig. S1 A, B). This result also proved to be consistent within the group of spines that was eliminated, as there did not appear to be a trend towards the elimination of small spines in particular (Fig. 3 A). Also, spines did not seem to be eliminated at specific times following the induction of LTD (Fig. S1 C). Not surprisingly, in control conditions conducted either in ACSF alone (n = 70 spines, 10 cells), in the presence of mGluR antagonists (n = 53 spines, 5 cells), in the absence of protein synthesis (n = 85 spines, 7 cells), or while activity was blocked (n = 52 spines, 6 cells), there was minimal variability between the initial and final size of spines (Fig. 3 B, C, D, E). These data demonstrate that the majority of spines are potentially capable of reducing or increasing their volume in response to synaptic activity.

### Spine Structural Changes following mGluR-LTD Last for 24 Hours

**Figure 4:**
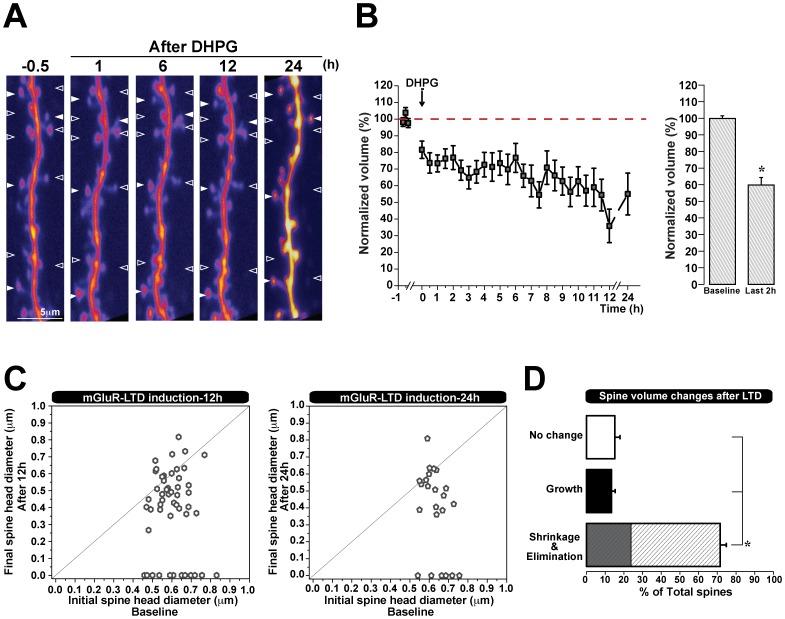
Spine shrinkage and elimination mediated by mGluR-LTD is long lasting. **A)** Representative two-photon images (maximum-intensity z-stack projections) of secondary dendrites from CA1 neurons transfected with Dendra-2C before and after LTD induction (DHPG). Z-stacks projections were collected once every 30 min for 12 h. Empty arrowheads indicate spines that shrink or eliminate and filled arrowheads indicate spines that do not change or grow 12 h and 24 h after LTD induction. **B**) Spine shrinkage was observed for over 24 hours following LTD induction. Normalization was performed as a percent of the average baseline volume of each spine (pooled data from 4 cells). **C**) Long lasting spine shrinkage and elimination are independent of initial spine size. Two-dimensional plot of initial *versus* final spine head diameter (µm) (15 min before and 12 h or 24 h after LTD). Spines which fall along the horizontal line do not show a change in volume. **D**) Quantification of spines that shrink, grow, or do not change after 12 h of mGluR-LTD induction from pooled data of 53 spines/4 cells. The shaded area within the shrinkage bar corresponds to the percentage of spines which are eliminated. *p<0.001. Error bars indicate means ± SEM.

We showed that spine shrinkage or elimination induced by mGluR-LTD lasts for 3 hours. As information can be stored for days or years [33], we wanted to test whether synaptic depression mediated structural changes in spines could also be very long lasting. Therefore, we induced mGluR LTD as before, but now followed spines continually for 12 hours, and acquired images once every 30 minutes. In some experiments, hippocampal slices were then returned to the incubator, and the same dendritic region was imaged 24 hours post-induction of plasticity. The images collected at 24 h showed an increased fluorescence intensity relative to earlier time points, likely due to their extended incubation time which allowed new fluorophores to accumulate without further photobleaching. We found that both at 12 h or 24 h following mGluR mediated synaptic depression, spines showed a significant reduction in volume (59.8±4.5% of initial spine volume at 12 h, n = 53 spines, 4 cells; 55.0±1.3% of initial spine volume at 24 h) (Fig. 4 A, B). Similarly to our previous findings, the majority of spines shrunk 12 hours after the induction of synaptic depression (72% of all spines decreased in volume), a proportion of which were also eliminated (24% of the spines which shrank), and as before, a few spines either grew or showed no change (13% grew, 15% did not change) (Fig. 4 D). It is unlikely that this spine loss following LTD is due to photo-damage, as we find that a proportion of spines either grows or does not change following the induction of plasticity. In accordance with our previous findings (Fig. 3 A), spine shrinkage and elimination remained independent of initial size when examined at longer time intervals of 12 h and 24 h (Fig. 4 C). These data show that the structural plasticity mediated by mGluR-LTD, namely the shrinkage and elimination of spines, can last at least 24 hours.

## Discussion

Depending on the type of activity, dendritic inputs can undergo bidirectional modifications of synaptic weights [21], [34], [35]. Therefore, in order to understand how neural circuits are shaped and maintained, it is important to understand how synaptic plasticity and structural connectivity are related. In the present study, we investigate the structural correlates of protein synthesis dependent long lasting synaptic depression. Using two-photon imaging of dendritic spines in hippocampal neurons, we find that the global induction of metabotropic glutamate receptor (mGluR) mediated LTD leads to significant and long lasting shrinkage and elimination of spines, for at least 24 hours. Similarly to mGluR mediated synaptic depression, we show that this form of structural plasticity also requires the production of new proteins. Furthermore, we determine that synaptic activity is necessary for the observed spine shrinkage, and we verify that this is not due to the indirect recruitment of NMDA receptor activation. Importantly, we find that both large and small spines can undergo such structural changes.

In order to alter dendritic connections, structural modifications should be able to occur at all inputs, suggesting that both large and small spines would potentially be affected. However, not all forms of synaptic plasticity are capable of inducing structural changes. For example, synaptic potentiation that does not require new protein synthesis is easier to induce at smaller spines [6], and likewise, smaller spines are more likely to shrink following NMDA mediated synaptic depression [20]. Conversely, strong potentiation leads to growth of spines of various sizes when new proteins are made [9]. Therefore, our findings demonstrating that both large and small spines can undergo shrinkage and also elimination following mGluR dependent LTD, highlights protein synthesis dependent synaptic plasticity as a potential mechanism by which to carry out synaptic remodeling.

When synapses are strengthened, the longevity of structural changes correlates with the longevity of the induced plasticity [9]. These varied kinetics may lead to significantly different plasticity outcomes, if for example, protein availability extends the window of time during which synapses may become bound together. Structural changes may also be more likely to occur in conjunction with this form of plasticity. However, it is unclear to what extent structural changes are associated with different forms of synaptic depression. The majority of studies have focused on NMDA receptor mediated LTD, which is not necessarily dependent on new protein synthesis [18]–[20]. We have begun to elucidate this relationship by demonstrating that the global induction of group I mGluR mediated LTD leads to robust shrinkage and elimination of spines. This brief, one-time induction of protein synthesis dependent plasticity leads to long lasting structural modifications, which we are able to follow for up to 24 hours. This finding was not expected, as the previously reported single application of the mGluR group I/II agonist resulted in synaptic depression, but no structural modifications [36]. Additionally, mGluR LTD at individual inputs leads to physiological depression at certain spines, but with no structural consequences reported [37]. It could be that in this latter case, the plasticity which resulted was not sufficient to activate local protein synthesis, and therefore no structural modifications were induced.

Since activation of group I mGluRs has been shown to increase neuronal excitability, and thus lead to depolarization and action potential discharge [38], [39], we investigated whether synaptic activity was necessary for mGluR mediated structural plasticity. Indeed, we find that activity is necessary for mGluR-LTD mediated spine shrinkage, as evidenced by the blockade of these changes in the presence of the sodium channel inhibitor TTX. Interestingly, L-type voltage dependent calcium channels (L-VDCCs) have been shown to interact with, and be facilitated by, mGluR5 [40]. Therefore, the TTX mediated blockade of action potentials, which trigger depolarization, will prevent mGluR facilitation of L-type VDCCs. Thus, interfering with a functional output of mGluRs, albeit indirectly, may inhibit the induction of plasticity and consequent structural changes.

Misregulation of mGluR plasticity has been implicated in mental retardation disorders, such as Fragile X syndrome [24]. Interestingly, this disorder has also been associated with altered spine density [41], and upregulated protein translation [42]. In accordance with this data, deletion of mGluR5 in mice leads to increased spine density in the cortex [27]. This points to the idea that mGluR plasticity has a structural output, although the direct nature of the regulation is still unclear. Our finding that mGluR-mediated LTD induces shrinkage and elimination of dendritic spines through a protein synthesis dependent mechanism, supports the idea that improper signaling through these receptors may lead to the abnormal spine pruning seen in mental retardation disorders.

Rewiring of neuronal contacts, via the clustering of synapses on a dendrite, would increase the storage capacity of a circuit, and provide a stable mechanism for effecting long-lasting changes [43]. In order for such remodeling to occur, certain inputs within a dendritic branch would be selectively potentiated and strengthened, while others would be selectively reduced or eliminated. Evidence indeed demonstrates that a subset of coactive inputs may be selectively strengthened following synaptic potentiation [9], although the counterpart of such plasticity during synaptic depression is unknown. Here we demonstrate the structural correlates of a protein synthesis dependent form of long-term depression (LTD) mediated by group I metabotropic glutamate receptors (mGluRs). Using two-photon imaging of dendritic spines in hippocampal neurons, we have shown that the global induction of mGluR-LTD leads to a significant and long lasting shrinkage and elimination of spines. We further determine that this form of structural plasticity requires the production of new proteins as well as synaptic activity. These findings demonstrate that indeed, bidirectional forms of plasticity correlate with bidirectional structural changes. Taken together, they provide a key mechanism by which to cluster synapses, allowing the pruning of specific inputs, based on the nature of the activity, and not necessarily depending on the initial size of the spines. These data also point to the dysregulation of mGluR signaling as a potential cause of the altered spine morphology seen in mental retardation disorders.

## Supporting Information

Figure S1 **Spine shrinkage and elimination are independent of initial spine volume.** **A**) Representative images of spines of various sizes that were quantified, from smaller at the bottom to larger at the top. **B**) Two-dimensional plot of initial *versus* final spine volumes per spine (µm^3^) (15 min before and 2 h after LTD). Spines which fall along the horizontal line do not change in volume during the experiment. **C**) Spine volume distribution for the group of spines that were eliminated following DHPG LTD. The graph shows that different sized spines were subject to elimination, and that they could be eliminated at various times following LTD stimulation.
(TIF)

## References

[1] Malenka RC, Bear MF (2004) LTP and LTD: an embarrassment of riches. Neuron 44: 5–21. 15450156 10.1016/j.neuron.2004.09.012

[2] Davis HP, Squire LR (1984) Protein synthesis and memory: a review. Psychol Bull 96: 518–559. 6096908

[3] Frey U, Krug M, Reymann KG, Matthies H (1988) Anisomycin, an inhibitor of protein synthesis, blocks late phases of LTP phenomena in the hippocampal CA1 region in vitro. Brain Res 452: 57–65. 3401749 10.1016/0006-8993(88)90008-x

[4] Maletic-Savatic M, Malinow R, Svoboda K (1999) Rapid dendritic morphogenesis in CA1 hippocampal dendrites induced by synaptic activity. Science 283: 1923–1927. 10082466 10.1126/science.283.5409.1923

[5] Engert F, Bonhoeffer T (1999) Dendritic spine changes associated with hippocampal long-term synaptic plasticity. Nature 399: 66–70. 10331391 10.1038/19978

[6] Matsuzaki M, Honkura N, Ellis-Davies GC, Kasai H (2004) Structural basis of long-term potentiation in single dendritic spines. Nature 429: 761–766. 15190253 10.1038/nature02617 PMC4158816

[7] Smith MA, Ellis-Davies GC, Magee JC (2003) Mechanism of the distance-dependent scaling of Schaffer collateral synapses in rat CA1 pyramidal neurons. J Physiol 548: 245–258. 12598591 10.1113/jphysiol.2002.036376 PMC2342790

[8] Fonseca R, Nagerl UV, Morris RG, Bonhoeffer T (2004) Competing for memory: hippocampal LTP under regimes of reduced protein synthesis. Neuron 44: 1011–1020. 15603743 10.1016/j.neuron.2004.10.033

[9] Govindarajan A, Israely I, Huang SY, Tonegawa S (2011) The dendritic branch is the preferred integrative unit for protein synthesis-dependent LTP. Neuron 69: 132–146. 21220104 10.1016/j.neuron.2010.12.008 PMC3032443

[10] Frey U, Morris RG (1998) Weak before strong: dissociating synaptic tagging and plasticity-factor accounts of late-LTP. Neuropharmacology 37: 545–552. 9704995 10.1016/s0028-3908(98)00040-9

[11] Harvey CD, Svoboda K (2007) Locally dynamic synaptic learning rules in pyramidal neuron dendrites. Nature 450: 1195–1200. 18097401 10.1038/nature06416 PMC3425382

[12] Collingridge GL, Peineau S, Howland JG, Wang YT (2010) Long-term depression in the CNS. Nat Rev Neurosci 11: 459–473. 20559335 10.1038/nrn2867

[13] Oliet SH, Malenka RC, Nicoll RA (1997) Two distinct forms of long-term depression coexist in CA1 hippocampal pyramidal cells. Neuron 18: 969–982. 9208864 10.1016/s0896-6273(00)80336-0

[14] Nicoll RA, Oliet SH, Malenka RC (1998) NMDA receptor-dependent and metabotropic glutamate receptor-dependent forms of long-term depression coexist in CA1 hippocampal pyramidal cells. Neurobiol Learn Mem 70: 62–72. 9753587 10.1006/nlme.1998.3838

[15] Gladding CM, Fitzjohn SM, Molnar E (2009) Metabotropic glutamate receptor-mediated long-term depression: molecular mechanisms. Pharmacol Rev 61: 395–412. 19926678 10.1124/pr.109.001735 PMC2802426

[16] Lujan R, Nusser Z, Roberts JD, Shigemoto R, Somogyi P (1996) Perisynaptic location of metabotropic glutamate receptors mGluR1 and mGluR5 on dendrites and dendritic spines in the rat hippocampus. Eur J Neurosci 8: 1488–1500. 8758956 10.1111/j.1460-9568.1996.tb01611.x

[17] Petralia RS, Sans N, Wang YX, Wenthold RJ (2005) Ontogeny of postsynaptic density proteins at glutamatergic synapses. Mol Cell Neurosci 29: 436–452. 15894489 10.1016/j.mcn.2005.03.013 PMC1414063

[18] Zhou Q, Homma KJ, Poo MM (2004) Shrinkage of dendritic spines associated with long-term depression of hippocampal synapses. Neuron 44: 749–757. 15572107 10.1016/j.neuron.2004.11.011

[19] Nagerl UV, Eberhorn N, Cambridge SB, Bonhoeffer T (2004) Bidirectional activity-dependent morphological plasticity in hippocampal neurons. Neuron 44: 759–767. 15572108 10.1016/j.neuron.2004.11.016

[20] Oh WC, Hill TC, Zito K (2012) Synapse-specific and size-dependent mechanisms of spine structural plasticity accompanying synaptic weakening. Proc Natl Acad Sci U S A. 10.1073/pnas.1214705110 PMC3557099 23269840

[21] Abbott LF, Nelson SB (2000) Synaptic plasticity: taming the beast. Nat Neurosci 3 Suppl: 1178–1183 10.1038/81453 11127835

[22] Huber KM, Kayser MS, Bear MF (2000) Role for rapid dendritic protein synthesis in hippocampal mGluR-dependent long-term depression. Science 288: 1254–1257. 10818003 10.1126/science.288.5469.1254

[23] Nosyreva ED, Huber KM (2005) Developmental switch in synaptic mechanisms of hippocampal metabotropic glutamate receptor-dependent long-term depression. J Neurosci 25: 2992–3001. 15772359 10.1523/JNEUROSCI.3652-04.2005 PMC6725134

[24] Bear MF, Huber KM, Warren ST (2004) The mGluR theory of fragile X mental retardation. Trends Neurosci 27: 370–377. 15219735 10.1016/j.tins.2004.04.009

[25] Kaufmann WE, Moser HW (2000) Dendritic anomalies in disorders associated with mental retardation. Cereb Cortex 10: 981–991. 11007549 10.1093/cercor/10.10.981

[26] McKinney BC, Grossman AW, Elisseou NM, Greenough WT (2005) Dendritic spine abnormalities in the occipital cortex of C57BL/6 Fmr1 knockout mice. Am J Med Genet B Neuropsychiatr Genet 136B: 98–102. 15892134 10.1002/ajmg.b.30183

[27] Chen CC, Lu HC, Brumberg JC (2012) mGluR5 knockout mice display increased dendritic spine densities. Neurosci Lett 524: 65–68. 22819970 10.1016/j.neulet.2012.07.014 PMC3727626

[28] Stoppini L, Buchs PA, Muller D (1991) A simple method for organotypic cultures of nervous tissue. J Neurosci Methods 37: 173–182. 1715499 10.1016/0165-0270(91)90128-m

[29] Bloodgood BL, Sabatini BL (2005) Neuronal activity regulates diffusion across the neck of dendritic spines. Science 310: 866–869. 16272125 10.1126/science.1114816

[30] Volk LJ, Daly CA, Huber KM (2006) Differential roles for group 1 mGluR subtypes in induction and expression of chemically induced hippocampal long-term depression. Journal of Neurophysiology 95: 2427–2438. 16421200 10.1152/jn.00383.2005

[31] Huber KM, Roder JC, Bear MF (2001) Chemical induction of mGluR5- and protein synthesis–dependent long-term depression in hippocampal area CA1. J Neurophysiol 86: 321–325. 11431513 10.1152/jn.2001.86.1.321

[32] Snyder EM, Philpot BD, Huber KM, Dong X, Fallon JR, et al (2001) Internalization of ionotropic glutamate receptors in response to mGluR activation. Nature Neuroscience 4: 1079–1085. 11687813 10.1038/nn746

[33] Mayford M, Siegelbaum SA, Kandel ER (2012) Synapses and memory storage. Cold Spring Harb Perspect Biol 4. 10.1101/cshperspect.a005751 PMC3367555 22496389

[34] Turrigiano GG (1999) Homeostatic plasticity in neuronal networks: the more things change, the more they stay the same. Trends Neurosci 22: 221–227. 10322495 10.1016/s0166-2236(98)01341-1

[35] Wang Z, Xu NL, Wu CP, Duan S, Poo MM (2003) Bidirectional changes in spatial dendritic integration accompanying long-term synaptic modifications. Neuron 37: 463–472. 12575953 10.1016/s0896-6273(02)01189-3

[36] Shinoda Y, Kamikubo Y, Egashira Y, Tominaga-Yoshino K, Ogura A (2005) Repetition of mGluR-dependent LTD causes slowly developing persistent reduction in synaptic strength accompanied by synapse elimination. Brain Res 1042: 99–107. 15823258 10.1016/j.brainres.2005.02.028

[37] Holbro N, Grunditz A, Oertner TG (2009) Differential distribution of endoplasmic reticulum controls metabotropic signaling and plasticity at hippocampal synapses. Proc Natl Acad Sci U S A 106: 15055–15060. 19706463 10.1073/pnas.0905110106 PMC2736455

[38] Brager DH, Johnston D (2007) Plasticity of intrinsic excitability during long-term depression is mediated through mGluR-dependent changes in I(h) in hippocampal CA1 pyramidal neurons. J Neurosci 27: 13926–13937. 18094230 10.1523/JNEUROSCI.3520-07.2007 PMC6673524

[39] Gereau RWt, Conn PJ (1995) Roles of specific metabotropic glutamate receptor subtypes in regulation of hippocampal CA1 pyramidal cell excitability. J Neurophysiol 74: 122–129. 7472317 10.1152/jn.1995.74.1.122

[40] Kato HK, Kassai H, Watabe AM, Aiba A, Manabe T (2012) Functional coupling of the metabotropic glutamate receptor, InsP3 receptor and L-type Ca2+ channel in mouse CA1 pyramidal cells. J Physiol 590: 3019–3034. 22586220 10.1113/jphysiol.2012.232942 PMC3406388

[41] Irwin SA, Patel B, Idupulapati M, Harris JB, Crisostomo RA, et al (2001) Abnormal dendritic spine characteristics in the temporal and visual cortices of patients with fragile-X syndrome: a quantitative examination. Am J Med Genet 98: 161–167. 11223852 10.1002/1096-8628(20010115)98:2<161::aid-ajmg1025>3.0.co;2-b

[42] Brown V, Jin P, Ceman S, Darnell JC, O’Donnell WT, et al (2001) Microarray identification of FMRP-associated brain mRNAs and altered mRNA translational profiles in fragile X syndrome. Cell 107: 477–487. 11719188 10.1016/s0092-8674(01)00568-2

[43] Govindarajan A, Kelleher RJ, Tonegawa S (2006) A clustered plasticity model of long-term memory engrams. Nat Rev Neurosci 7: 575–583. 16791146 10.1038/nrn1937

