# Supplementary figures and images for: Long Lasting Protein Synthesis- and Activity-Dependent Spine Shrinkage and Elimination after Synaptic Depression
Source: PLoS One. 2013 Aug 9;8(8):e71155. doi: 10.1371/journal.pone.0071155 (PMC3739806; doi:10.1371/journal.pone.0071155)

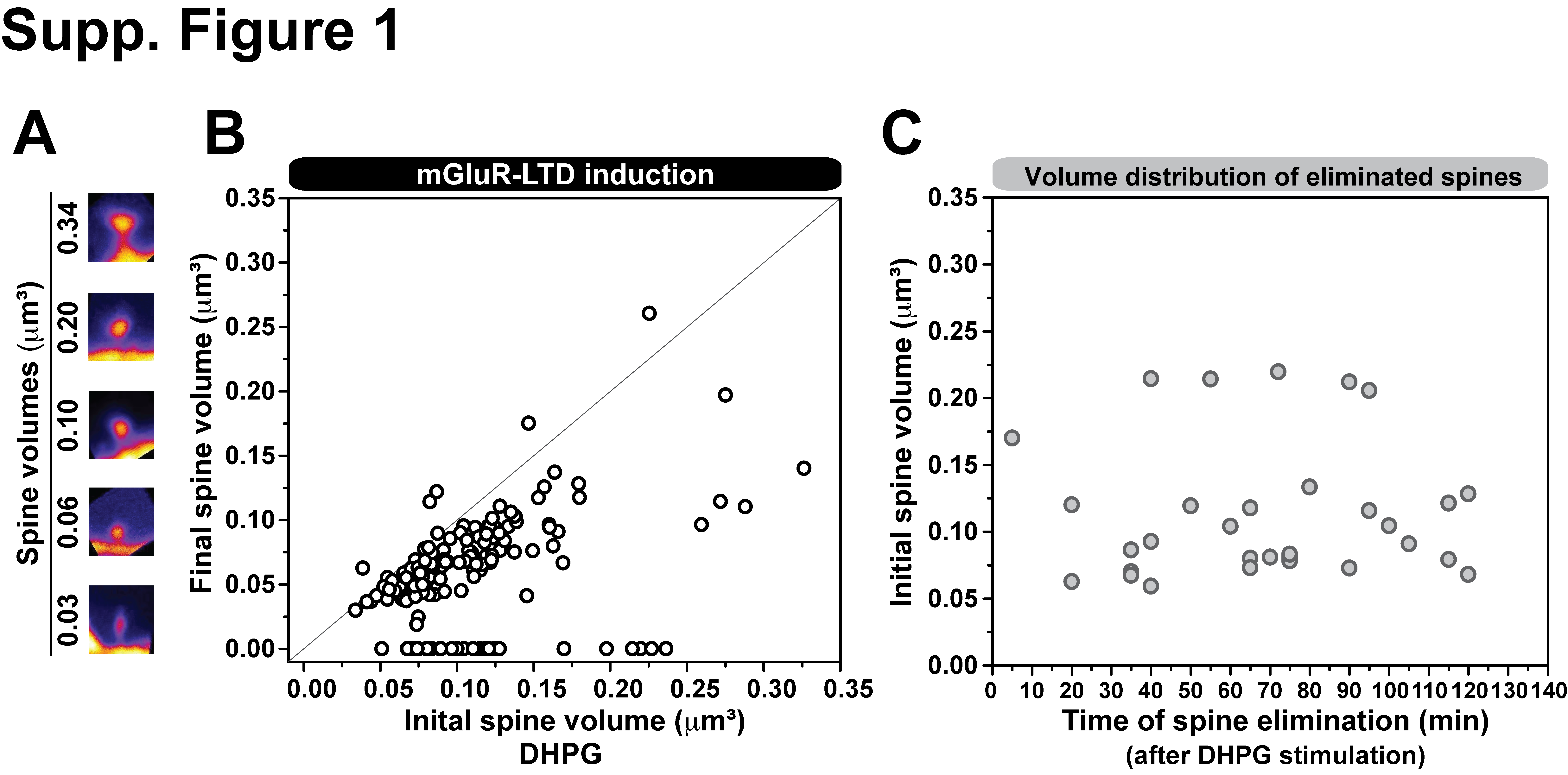

Supplement: Figure S1 — Spine shrinkage and elimination are independent of initial spine volume. A) Representative images of spines of various sizes that were quantified, from smaller at the bottom to larger at the top. B) Two-dimensional plot of initial versus final spine volumes per spine (µm3) (15 min before and 2 h after LTD). Spines which fall along the horizontal line do not change in volume during the experiment. C) Spine volume distribution for the group of spines that were eliminated following DHPG LTD. The graph shows that different sized spines were subject to elimination, and that they could be eliminated at various times following LTD stimulation. (TIF) [file pone.0071155.s001.tif]
